# A systematic review of the literature assessing the outcomes of stapled haemorrhoidopexy versus open haemorrhoidectomy

**DOI:** 10.1007/s10151-020-02314-6

**Published:** 2020-10-24

**Authors:** Q. Z. Ruan, W. English, A. Hotouras, C. Bryant, F. Taylor, S. Andreani, S. D. Wexner, S. Banerjee

**Affiliations:** 1 Whipps Cross University Hospital, Barts Health NHS Trust, London, UK; 2 National Bowel Research Centre, Blizard Institute, QMUL, 2 Newark Street, London, E1 2AT UK; 3 Cleveland Clinic Florida, Fort Lauderdale, FL USA; 4 Barking, Havering and Redbridge University Hospitals NHS Trust, Romford, UK

**Keywords:** Haemorrhoidectomy, Stapled, Open, Outcomes

## Abstract

**Background:**

Symptomatic haemorrhoids affect a large number of patients throughout the world. The aim of this systematic review was to compare the surgical outcomes of stapled haemorrhoidopexy (SH) versus open haemorrhoidectomy (OH) over a 20-year period.

**Methods:**

Randomized controlled trials published between January 1998 and January 2019 were extracted from Pubmed using defined search criteria. Study characteristics and outcomes in the form of short-term and long-term complications of the two techniques were analyzed. Any changes in trend of outcomes over time were assessed by comparing article groups 1998–2008 and 2009–2019.

**Results:**

Twenty-nine and 9 relevant articles were extracted for the 1998–2008 (period 1) and 2009–2019 (period 2) cohorts, respectively. Over the two time periods, SH was found to be a safe procedure, associated with statistically reduced operative time (in 13/21 studies during period 1 and in 3/8 studies during period 2), statistically less intraoperative bleeding (3/7 studies in period 1 and 1/1 study in period 2) and consistently less early postoperative pain on the visual analogue scale (12/15 studies in period 1 and 4/5 studies in period 2) resulting in shorter hospital stay (12/20 studies in period 1 and 2/2 studies in period 2) at the expense of a higher cost. In the longer term, although chronic pain in SH and OH patents is comparable, patient satisfaction with SH may decline with time and at 2-year follow-up OH appeared to be associated with greater patient satisfaction.

**Conclusions:**

SH appears to be safe with potential advantages, at least in the short term, but the evidence is lacking at the moment to suggest its routine use in clinical practice.

## Introduction

Symptomatic haemorrhoids account for approximately 3.3 million outpatient encounters annually in the United States [1], while up to 37% of the general population in the United Kingdom may be affected by the same disease process [2]. Grade III and IV haemorrhoidal disease (Goligher classification) responds more favourably to surgical treatment [3]. Traditional open haemorrhoidectomy (OH) is still the gold standard operation but it is associated with significant postoperative pain and a small risk of injury to the anal sphincter complex [4, 4]. Novel surgical procedures such as the haemorrhoidal artery ligation operation (HALO) and stapled haemorrhoidopexy (SH) have been increasingly used in recent years. However, robust evidence strongly supportive of a specific technique is lacking. The introduction of SH in 1998 promptly caught the interest of colorectal surgeons. The technique has been used mostly in North America and European countries. The United Kingdom has yet to adopt this procedure on a significant scale. The aim of this systematic review was to assess the surgical outcomes of this procedure in comparison to OH over a 20-year period (since the introduction of SH in 1998) and assess changes in its safety profile to the present day.

## Materials and methods

### Search strategy and study selection

The PubMed database was searched for relevant studies published between January 1998 and January 2019. The search criteria ‘staple* AND haemorrhoid* OR hemorrhoid’ were broadly used and a series of rigid inclusion criteria were subsequently applied. A study was deemed suitable for inclusion if the publication [1] was a randomized controlled trial (RCT) [2], compared at least two surgical methods of haemorrhoidal management with mandatory inclusion of OH [3], involved human subjects, and [4] was written in English. Two independent reviewers (QZR and AH) used the above-mentioned inclusion criteria for all research papers derived from the search. Studies were included after titles and abstracts were evaluated for suitability. Articles without abstracts were excluded. Full-text versions were then acquired. In the event of disagreement, a consensus method was used amongst the two reviewers The review was conducted in accordance with the guidelines set out in the “Preferred Reporting Items for Systematic Reviews and Meta-Analyses” (PRISMA) statement [6]. Collectively, selected articles were subcategorized into two classes by year (1998–2008; 2009–2019) for data interpretation and subsequent comparison.

### Data extraction and outcome measures

Data extracted included study characteristics (study objective, type of study, methods of analysis) and outcomes (immediate complications, long-term complications, and overall qualitative conclusion). The derivative qualitative conclusions were defined by the positions represented by the most number of articles for that category (SH superior, SH similar to OH, SH inferior). If there were equal numbers of articles for opposing positions, the position with the most number of articles with supporting *p* values was taken as the overall qualitative conclusion. Only statistically significant *p* values were taken as the benchmark when assessing validity of study conclusions. Some studies did not calculate statistical significance when comparing the above parameters and chose to qualitatively summarize their data. We have made it a point to label their *p* values ‘undefined’, but their observations continued to be acknowledged during our analyses.

### Statistical analysis

GraphPad Prism 7.0 (GraphPad Software, Inc. La Jolla, CA, USA) was used for all statistical calculations in this paper. Student’s t test and the Mann–Whitney test were employed to compare continuous trends in complications across the two chronological classes (1998–2008 and 2009–2019), while Fisher’s exact test was used to determine differences in categorical outcomes. A *p* value < 0.05 was considered statistically significant.

## Results

**Fig. 1:**
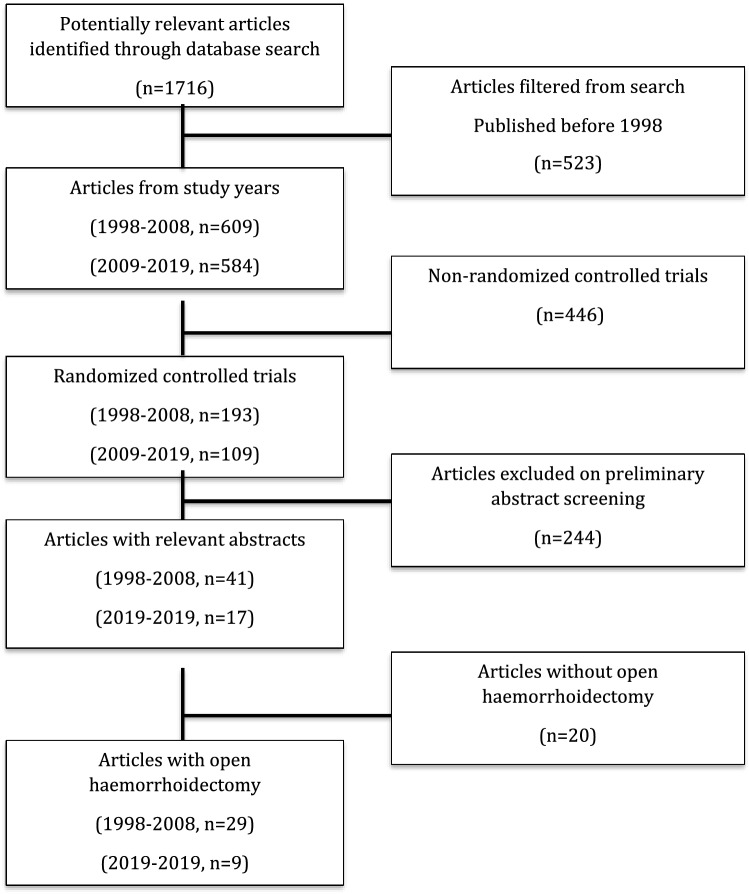
PRISMA study inclusion flowchart

In total, 1716 articles were initially identified by the search on 12th January 2019. After abstract screening and exclusion, a total of 38 articles met inclusion criteria for further analysis (Fig. 1).

Twenty-nine [7–35] and 9 articles [36–44] were selected for the chronological categories of 1998–2008 and 2009–2019, respectively. The mean number of patients per study was 197 ± 80 (range 22–3000 patients). Patients were followed-up for a mean of 76.5 ± 14.4 weeks (range 4.4–365.4) weeks. Randomisation was conducted prior to treatment for the majority of the studies (24 of 38). Twenty-three of the studies demonstrated that there was no statistically significant difference in demographics between the patient groups being compared. Basic study characteristics are demonstrated in Tables 1, 2.

**Table 1:** Basic study characteristics from 1998 to 2008 cohort (*n* = 29 papers)

Year	Journal	Author	Study size (*n*)	Haemorrhoid grades included	Techniques investigated	Defined SH equipment	Mean follow up (weeks)	Defined comparable demographics	Defined randomization methods	Cost analyses	Patient satisfaction measurement
2000	Lancet	Mehigan et al.	40	III, IV	SH vs OH	CDH 33 or HCS 33	10	Yes	Yes	No	Yes
2000	Lancet	Rowsell et al.	22	III	SH vs OH	CDH or PPH	6	Yes	Yes	No	No
2001	Colorectal Dis	Brown et al.	35	Undefined	SH vs OH	PPH	6	Yes	Yes	No	No
2001	Br J Surg	Ganio et al.	100	III, IV	SH vs OH	ILS 33	4.35	Yes	Yes	No	Yes
2001	Br J Surg	Shalaby et al.	200	Undefined	SH vs OH	HCS 33	52.2	Yes	Yes	No	No
2002	Arch Surg	Hetzer et al.	40	II, III	SH vs OH	HCS 33	52.2	Yes	No	No	No
2002	Tech Coloproctol	Ooi et al.	119	Undefined	SH vs OH	Undefined	73.95	No	No	No	Yes
2002	Br J Surg	Ortiz et al.	31	IV	SH vs OH	PPH	52.2	Yes	Yes	No	Yes
2002	Int J Colorectal Dis	Pavlidis et al.	80	II, III, IV	SH vs OH	PPH	13.05	Yes	No	No	Yes
2002	Dis Colon Rectum	Wilson et al.	89	III	SH vs OH	PPH or Autosuture	8	Yes	Yes	Yes	Yes
2003	Dis Colon Rectum	Cheetham et al.	31	Undefined	SH vs OH	PPH	26.1	Yes	Yes	No	Yes
2003	Dis Colon Rectum	Kairaluoma et al.	60	III	SH vs OH	PPH	52.2	No	No	No	Yes
2003	World J Surg	Palimento et al.	52	III, IV	SH vs OH	PPH	26.1	Yes	Yes	No	Yes
2003	Lancet	Smyth et al.	36	Undefined	SH vs OH	Undefined	156.6	No	No	No	Yes
2004	Colorectal Dis	Au-Yong et al.	22	Undefined	SH vs OH	Undefined	182.7	No	No	No	No
2004	Hong Kong Med J	Lau et al.	24	II, III	SH vs OH	PPH	8	Yes	Yes	No	No
2004	Int J Colorectal Dis	Racalbuto et al.	100	III, IV	SH vs OH	CDH 33	208.8	No	Yes	No	Yes
2005	Surg Endosc	Basdanis et al.	95	III, IV	LH vs OH	Undefined	104.4	No	Yes	Yes	No
2005	Am J Surg	Bikhchandani et al.	84	III, IV	SH vs OH	Undefined	52.2	Yes	No	No	Yes
2005	Dis Colon Rectum	Chung et al.	88	III	SH vs OH	PPH	26.1	Yes	Yes	No	Yes
2005	Ann Surg	Gravie et al.	134	Undefined	SH vs OH	PPH	104.4	Yes	Yes	No	Yes
2005	Dis Colon Rectum	Kraemer et al.	50	III, IV	LH vs HSH	PPH	6	No	Yes	No	Yes
2006	Int J Colorectal Dis	Picchio et al.	74	Undefined	SH vs OH	Undefined	269.7	No	No	No	Yes
2007	Br J Surg	Ganio et al.	80	Undefined	SH vs OH	PPH	365.4	No	No	No	No
2007	J Gastrointest Surg	Lai et al.	80	I, II, III, IV	SH vs OH	PPH	26.1	Yes	No	No	Yes
2007	Colorectal Dis	Martinsons et al.	480	III, IV	SH vs OH/ SH plus	PPH	26.1	Yes	No	No	Yes
2007	Dis Colon Rectum	Wong et al.	41	Undefined	SH vs OH	PPH	12	Yes	Yes	No	Yes
2008	ANZ J Surg	Leventoglu et al.	60	III, IV	SH vs OH	PPH	17.4	Yes	No	No	Yes
2008	J Gastrointest Surg	Stolfi et al.	100	III, IV	SH vs OH	PPH	26.1	Yes	Yes	No	Yes

*CH* closed haemorrhoidectomy, *HAL* haemorrhoidal artery ligation, *HSH* harmonic scalpel haemorrhoidectomy, *LH* Ligasure haemorrhoidectomy, *OH* open haemorrhoidectomy, *RL* rubberband ligation, *SCH* semi-closed haemorrhoidectomy, *SH* stapled haemorrhoidectomy, *PPH* procedure for prolapse and haemorrhoids, *CDH* curved intraluminal stapler, *HCS* haemorrhoidal circular stapler

**Table 2:** Basic study characteristics from 2009 to 2019 cohort (n = 9 papers)

Year	Journal	Author	Study size (*n*)	Haemorrhoid grades included	Techniques investigated	Defined SH equipment	Mean follow up (weeks)	Defined comparable demographics	Defined randomization methods	Cost analyses	Patient satisfaction measurement
2010	Br J Surg	Nystrom et al.	207	III, IV	SH vs OH	PPH	52.2	No	Yes	No	No
2012	G Chir	Ammaturo et al.	80	III	SH vs OH	PPH	104.4	Yes	Yes	No	Yes
2012	Surg Laparosc Endosc Percutan Tech	Arslani et al.	98	III	SH vs LH	Undefined	104.4	Yes	Yes	No	No
2013	J Gastrointest Surg	Kim et al.	130	III	SH vs OH	PPH	313.2	Yes	Yes	No	No
2015	World J Gastroenterol	Wang et al.	480	III, IV	SH vs OH	TST	52.2	Yes	No	No	Yes
2015	Dis Colon Rectum	Ripetti et al.	180	III, IV	SH vs OH vs SCH	Undefined	17.4	Yes	Yes	No	No
2015	Asian J Surg	Bilgin et al.	99	III, IV	SH vs HSH	PPH	156.6	Yes	No	No	No
2016	Lancet	Watson et al.	777	II, III, IV	SH vs OH/CH	Undefined	104.4	No	Yes	Yes	Yes
2017	Surg Innov	He et al.	3000	II, III, IV	SH vs OH vs RL	PPH	26.1	Yes	Yes	No	No

*CH* closed haemorrhoidectomy, *HAL* haemorrhoidal artery ligation, *HSH* harmonic scalpel haemorrhoidectomy, *LH* Ligasure haemorrhoidectomy, *OH* open haemorrhoidectomy, *RL* rubberband ligation, *SCH* semi-closed haemorrhoidectomy, *SH* stapled haemorrhoidectomy, *PPH* procedure for prolapse and haemorrhoids, *CDH* curved intraluminal stapler; *HCS* haemorrhoidal circular stapler, *TST* tissue selective therapy stapler

Fourteen of the trials included procedures on grades III and IV haemorrhoids only. The remaining trials included interventions on grades II, III and IV haemorrhoids in various permutations. Ten articles did not ascertain the types of haemorrhoids treated. All patients had previously failed non-operative management. Specifically for SH, the majority of studies used the “Procedure for Prolapse and Haemorrhoids” (PPH) stapler (22 of 38). All studies assessed similar outcomes, classifying them largely into immediate complications, long-term complications, recurrences and patient satisfaction. Three of the trials performed cost analyses.

For comparisons against SH, alternative operative interventions considered OH Harmonic scalpel haemorrhoidectomy (HSH) and Ligasure haemorrhoidectomy (LH).

Studies performed from 2009 to 2019 incorporated larger study populations (561 ± 314 vs 84 ± 16, *p* = 0.009) and trended towards a longer follow-up period (103.4 ± 30.2 weeks vs 68.1 ± 16.4 weeks, *p* = 0.3) compared to the period of 1998–2008. The likelihood of the RCTs having formal descriptions of comparable patient demographics (*p* > 0.9) and study randomization (*p* = 0.44) were similar over the two time periods, as were studies to include a section on cost analyses comparing the methods of haemorrhoidectomy (*p* > 0.9).

### Immediate complications

Multiple immediate complications and outcomes were assessed among various operative groups including prolonged operative time, intraoperative blood loss, postoperative burning, postoperative bleeding, urinary retention, length of hospital stay and wound infection.

From 1998 to 2008, a total of 13 out of 21 studies reported a significantly shorter operative duration for SH than for OH in comparison to just 2 which stated otherwise (Table 3). Similarly from 2009 to 2019 (Table 4), five of eight studies reported similar findings (3 to statistical significance) with just one arguing the reverse (that SH requires longer operative time). Immediate postoperative bleeding was similar between SH and OH in both decade-long categories but more studies revealed SH to be favourable in reducing urinary retention in the 1998–2008 group (10 of 17 vs 4 of 17), although this only reached significance in 1 study, as well as the 2009–2019 group (3 of 6 vs 0 of 6) 2 studies reaching significance. Total length of hospital stay of SH patients was shorter in both groups, with 14 of 20 (10 reaching significance) (1998–2008) and 3 of 6 (2 reaching significance) (2009–2019) articles demonstrating shorter hospital stay over the last 20 years. The above data are summarized in Tables 3, 4.

**Table 3:** Risks of short-term complications (articles published from 1998 to 2008)

Complication type	Studies which assessed defined complication	Study conclusions	*p* value
Operative time	Basdanis et al.	SH longer operative time vs OH	< 0.05
	Brown et al.	SH longer operative time vs OH	< 0.05
	Lau et al.	SH longer operative time vs OH	0.26
	Chung et al.	SH shorter operative time vs HSH	0.52
	Bikhchandani et al.	SH shorter operative time vs OH	< 0.001
	Gravie et al.	SH shorter operative time vs OH	0.035
	Hetzer et al.	SH shorter operative time vs OH	< 0.001
	Kairaluoma et al.	SH shorter operative time vs OH	0.49
	Kraemer et al.	SH shorter operative time vs OH	0.1858
	Lai et al.	SH shorter operative time vs OH	< 0.01
	Leventoglu et al.	SH shorter operative time vs OH	0.0001
	Martinsons et al.	SH shorter operative time vs OH	0.001
	Ortiz et al.	SH shorter operative time vs OH	0.001
	Palimento et al.	SH shorter operative time vs OH	0.041
	Pavlidis et al.	SH shorter operative time vs OH	< 0.05
	Racalbuto et al.	SH shorter operative time vs OH	0.164
	Shalaby et al.	SH shorter operative time vs OH	< 0.001
	Wilson et al.	SH shorter operative time vs OH	< 0.001
	Rowsell et al.	SH similar operative time vs OH	Undefined
	Stolfi et al.	SH similar operative time vs OH	0.94
	Wong et al.	SH similar operative time vs OH	0.6
Intraop blood loss	Bikhchandani et al.	SH less blood loss vs OH	< 0.001
	Chung et al.	SH less blood loss vs OH	0.57
	Wilson et al.	SH less blood loss vs OH	< 0.001
	Wong et al.	SH less blood loss vs OH	0.58
	Basdanis et al.	SH more blood loss vs OH	< 0.05
	Palimento et al.	SH more blood loss vs OH	0.5
	Brown et al.	SH similar blood loss vs OH	Undefined
Postop bleeding	Brown et al.	SH less blood loss vs OH	< 0.05
	Cheetham et al.	SH less blood loss vs OH	0.17
	Kraemer et al.	SH less blood loss vs OH	Undefined
	Lai et al.	SH less blood loss vs OH	1
	Leventoglu et al.	SH less blood loss vs OH	0.017
	Ortiz et al. (2002)	SH less blood loss vs OH	Undefined
	Shalaby et al.	SH less blood loss vs OH	Undefined
	Stolfi et al.	SH less blood loss vs OH	< 0.001
	Basdanis et al.	SH more blood loss vs OH	0.5
	Gravie et al.	SH more blood loss vs OH	0.477
	Hetzer et al.	SH more blood loss vs OH	Undefined
	Kairaluoma et al.	SH more blood loss vs OH	Undefined
	Palimento et al.	SH more blood loss vs OH	1
	Pavlidis et al.	SH more blood loss vs OH	Undefined, ns
	Racalbuto et al.	SH more blood loss vs OH	Undefined
	Wilson et al.	SH more blood loss vs OH	Undefined
	Ganio et al.	SH similar blood loss vs OH	Undefined, ns
	Martinsons et al.	SH similar blood loss vs OH	0.809
Postop urinary retention	Bikhchandani et al.	SH less retention vs OH	Undefined
	Gravie et al.	SH less retention vs OH	0.62
	Hetzer et al.	SH less retention vs OH	Undefined
	Lau et al.	SH less retention vs OH	Undefined
	Leventoglu et al.	SH less retention vs OH	0.017
	Ortiz et al. (2002)	SH less retention vs OH	Undefined
	Palimento et al.	SH less retention vs OH	0.54
	Racalbuto et al.	SH less retention vs OH	Undefined
	Shalaby et al.	SH less retention vs OH	Undefined
	Wong et al.	SH less retention vs OH	0.48
	Chung et al.	SH more retention vs HSH	Undefined, ns
	Basdanis et al.	SH more retention vs OH	< 0.5
	Stolfi et al.	SH more retention vs OH	Undefined
	Wilson et al.	SH more retention vs OH	Undefined
	Lai et al.	SH similar retention vs OH	1
	Martinsons et al.	SH similar retention vs OH	0.243
	Mehigan et al.	SH similar retention vs OH	Undefined
Length of hospital stay	Stolfi et al.	SH longer length of stay vs OH	0.014
	Chung et al.	SH shorter length of stay vs HSH	0.02
	Bikhchandani et al.	SH shorter length of stay vs OH	< 0.01
	Ganio et al.	SH shorter length of stay vs OH	0.01
	Gravie et al.	SH shorter length of stay vs OH	< 0.001
	Hetzer et al.	SH shorter length of stay vs OH	0.17
	Lai et al.	SH shorter length of stay vs OH	< 0.01
	Lau et al.	SH shorter length of stay vs OH	0.014
	Martinsions et al.	SH shorter length of stay vs OH	0.001
	Pavlidis et al.	SH shorter length of stay vs OH	< 0.05
	Racalbuto et al.	SH shorter length of stay vs OH	0.098
	Rowsell et al.	SH shorter length of stay vs OH	< 0.001
	Shalaby et al.	SH shorter length of stay vs OH	< 0.001
	Wilson et al.	SH shorter length of stay vs OH	Undefined, ns
	Wong et al.	SH shorter length of stay vs OH	0.16
	Basdanis et al.	SH similar length of stay vs OH	Undefined
	Brown et al.	SH similar length of stay vs OH	Undefined
	Kairaluoma et al.	SH similar length of stay vs OH	0.1
	Kraemer et al.	SH similar length of stay vs OH	Undefined
	Mehigan et al.	SH similar length of stay vs OH	0.05

*OH* open haemorrhoidectomy, *SH* stapled haemorrhoidectomy, *HSH* harmonic scalpel haemorrhoidectomy

**Table 4:** Risks of short-term complications in articles published from 2009 to 2019

Complication type	Studies which assessed defined complication	Study conclusions	*p* value
Operative time	He et al.	SH shorter intraop time vs OH	< 0.05
	Kim et al.	SH shorter intraop time vs OH	< 0.001
	Ripetti et al.	SH shorter intraop time vs OH	Undefined
	Wang et al.	SH shorter intraop time vs OH	< 0.001
	Ammaturo et al.	SH shorter intraop time vs OH	Undefined
	Nystrom et al.	SH similar intraop time vs OH	0.247
	Watson et al.	SH similar intraop time vs THD	Undefined
	Bilgin et al.	SH longer intraop time vs HSH	Undefined
Intraop blood loss	Wang et al.	SH less blood loss vs OH	< 0.001
Postop burning sensation	Kim et al.	SH less symptomatic than OH	< 0.001
Postop bleeding	Ammaturo et al.	SH less blood loss vs OH	Undefined
	Nystrom et al.	SH less blood loss vs OH	Undefined
	Arslani et al.	SH similar blood loss vs OH	0.504
	Kim et al.	SH similar blood loss vs OH	Undefined
	Ripetti et al.	SH similar blood loss vs OH/SCH	0.21
	Bilgin et al.	SH more blood loss vs HSH	Undefined
	He et al.	SH more blood loss vs OH	< 0.05
	Watson et al.	SH more blood loss vs OH/CH	Undefined
Postop urinary retention	Ammaturo et al.	SH less retention risk vs OH	Undefined
	He et al.	SH less retention risk vs OH	< 0.05
	Wang et al.	SH less retention risk vs OH	0.001
	Arslani et al.	SH similar retention risk vs OH	0.898
	Kim et al.	SH similar retention risk vs OH	1
	Ripetti et al.	SH similar retention risk vs OH, SCH	0.2
Length of hospital stay	Ammaturo et al.	SH shorter length of stay vs OH	Undefined
	He et al.	SH shorter length of stay vs OH	< 0.05
	Wang et al.	SH shorter length of stay vs OH	< 0.01
	Bilgin et al.	SH similar length of stay vs HSH	Undefined
	Nystrom et al.	SH similar length of stay vs OH	0.456
	Watson et al.	SH similar length of stay vs OH/CH	Undefined
Wound infection	Ammaturo et al.	SH similar infection risk vs OH	Undefined
	Watson et al.	SH similar infection risk vs OH/CH	Undefined

*CH* closed haemorrhoidectomy, *HSH* harmonic scalpel haemorrhoidectomy, *OH* open haemorrhoidectomy, *SCH* semi-closed haemorrhoidectomy, *SH* stapled haemorrhoidectomy, *THD* transanal haemorrhoidal dearterialization

A total of 20 studies used a visual analogue scale (VAS) to assess postoperative pain. Earlier studies (1998–2008) tended to measure postoperative pain over the short term (hours to days), with only one study exploring pain at the 4-week mark [34]. These studies were consistent in demonstrating significantly lower VAS scores in the SH group, especially in the hours to days following surgery. More recent studies performed after 2008 explored pain control up to a year post procedure [47]. The advantages of SH in limiting short-term postoperative pain in this group largely corroborate with findings in the 1998–2008 group, while long-term benefits were more difficult to discern (Table 5).

**Table 5:** Visual analogue scale for pain as demonstrated by individual studies

Visual analogue scale (VAS)				
Author	Postoperative duration at pain measurement	SH	CH/HAL/HSH/OH/SCH/SH	*p* value
1998–2008				
Basdanis et al.	1 day	5	7	< 0.001
	1 week	1	2	Undefined
Bikhchandani et al.	12 h	3.45	4.86	< 0.001
	1 day	3.64	6.36	< 0.001
	3 days	1.52	4.5	< 0.001
	1 week	0.57	2.31	< 0.01
	15 days	0.21	1.05	< 0.001
Cheetham et al.	10 days	4.5	9	0.018
Chung et al.	7 days	1.5	3.5	0.002
Gravie et al.	10 days	Undefined	Undefined	< 0.001 (in favour of SH)
Hetzer et al.	1 day	2.7	6.3	< 0.01
	2 days	1.7	6.3	< 0.01
	3 days	0.8	5.4	< 0.01
	4 days	0.5	4.8	< 0.01
Kraemer et al.	3 weeks	Undefined	Undefined	0.99
Lai et al.	1 day	3.53	7.18	< 0.01
	7 days	1.98	3.68	< 0.01
	2 weeks	1.33	1.85	< 0.01
Lau et al.	2 days	4	3.1	0.93
Leventoglu et al.	8 h	5.7	7.75	0.0001
	1 day	1.3	4.5	0.0001
	2 days	0.9	3	0.025
	7 days	0.15	1.5	0.026
	2 weeks	0	1	0.014
	4 weeks	0	1	0.015
Palimento et al.	4 h	Undefined	Undefined	< 0.001 (in favour of SH)
Pavlidis et al.	3 h	2.5	3.4	< 0.05
	6 h	2.9	3.9	< 0.05
	12 h	2.3	3.6	< 0.05
	1 day	0.7	2.4	< 0.01
Shalaby et al.	1 day	2.5	7.6	< 0.001
	1 week	0.4	2.6	< 0.001
Stolfi et al.	2 days	5.11	5.13	0.96
	8 days	3.98	4.82	0.016
Wong et al.	1 week	4.1	5.7	0.02
2009–2019				
Kim et al.	1 week	3.1	6.2	< 0.001
	2 weeks	0.5	3	< 0.001
	4 weeks	0.05	0.6	< 0.001
Lehur et al.	2 weeks	2.8	2.2	0.03
Leung et al.	1 week	3.7	3.4	0.09
	2 months	1	1	0.2
	4 months	1	1	0.079
	1 year	1	1	0.767
Wang et al.	12 h	5.1	7.2	< 0.001
Watson et al.	1 week	4	5.3	< 0.0001
	3 weeks	1.8	2.6	0.0026
	6 weeks	1.3	1.3	0.96

*CH* closed haemorrhoidectomy, *HAL* haemorrhoidal artery ligation, *HSH* harmonic scalpel haemorrhoidectomy, *OH* open haemorrhoidectomy, *SCH* semi-closed haemorrhoidectomy, *SH* stapled haemorrhoidectomy, *THD* transanal haemorrhoidal dearterialization

### Long-term complications

The long-term complications evaluated included fistulae, incontinence, anal stenosis, tenesmus, chronic pain and recurrence (Tables 6, 7). There was limited evidence of any single complication being significantly more closely associated with SH compared to the other surgical approaches. Risks of incontinence and recurrence were the two complications most frequently measured by studies throughout the last 2 decades.

**Table 6:** Risks of long-term complications in articles published in 1998–2008

Complication type	Studies which assessed defined complication	Study conclusions	*p* value
Fistulae	Ortiz et al.	SH lower risk of fistula formation vs OH	Undefined
Incontinence	Bikhchandani et al.	SH lower risk of incontinence vs OH	Undefined
	Mehigan et al.	SH lower risk of incontinence vs OH	Undefined
	Pavlidis et al.	SH lower risk of incontinence vs OH	Undefined
	Au Yong et al.	SH similar risk of incontinence vs OH	0.56
	Ganio et al.	SH similar risk of incontinence vs OH	0.479
	Gravie et al.	SH similar risk of incontinence vs OH	0.29
	Hetzer et al.	SH similar risk of incontinence vs OH	Undefined
	Kairaluoma et al.	SH similar risk of incontinence vs OH	0.61
	Kraemer et al.	SH similar risk of incontinence vs OH	Undefined
	Leventoglu et al.	SH similar risk of incontinence vs OH	0.114
	Smyth et al.	SH similar risk of incontinence vs OH	0.409
Anal stenosis	Au Yong et al.	SH similar risk of stenosis vs OH	Undefined
	Bikhchandani et al.	SH similar risk of stenosis vs OH	Undefined
	Brown et al.	SH similar risk of stenosis vs OH	ns
	Ganio et al.	SH similar risk of stenosis vs OH	Undefined
	Gravie et al.	SH similar risk of stenosis vs OH	1
	Martinsons et al.	SH similar risk of stenosis vs OH	0.663
	Shalaby et al.	SH similar risk of stenosis vs OH	Undefined
Chronic pain	Bikhchandani et al.	SH lower risk of pain vs OH	Undefined
	Brown et al.	SH lower risk of pain vs OH	< 0.05
	Kraemer et al.	SH lower risk of pain vs OH	Undefined
	Martinsons et al.	SH lower risk of pain vs OH	< 0.001
	Kairaluoma et al.	SH similar risk of pain vs OH	1
	Ooi et al.	SH similar risk of pain vs OH	Undefined
	Picchio et al.	SH similar risk of pain vs OH	1
Tenesmus/ Urgency	Ganio et al.	SH higher risk of urgency vs OH	Undefined
	Gravie et al.	SH similar risk of tenesmus vs OH	1
	Au Yong et al.	SH similar risk of urgency vs OH	0.41
	Mehigan et al.	SH similar risk of urgency vs OH	Undefined
	Ortiz et al.	SH similar risk of urgency vs OH	Undefined
	Smyth et al.	SH similar risk of urgency vs OH	undefined
	Stolfi et al.	SH similar risk of urgency vs OH	Undefined
Recurrence	Basdanis et al.	SH higher risk of recurrence vs OH	Undefined
	Bikhchandani et al.	SH higher risk of recurrence vs OH	Undefined
	Ganio et al.	SH higher risk of recurrence vs OH	0.001
	Ortiz et al.	SH higher risk of recurrence vs OH	0.004
	Racalbuto	SH higher risk of recurrence vs OH	Undefined
	Wong et al.	SH lower risk of recurrence vs OH	0.002
	Chung et al.	SH similar risk of recurrence vs HSH	0.93
	Au Yong et al.	SH similar risk of recurrence vs OH	0.57
	Cheetham et al.	SH similar risk of recurrence vs OH	ns
	Ganio et al.	SH similar risk of recurrence vs OH	0.562
	Gravie et al.	SH similar risk of recurrence vs OH	0.498
	Ooi et al.	SH similar risk of recurrence vs OH	ns
	Shalaby et al.	SH similar risk of recurrence vs OH	Undefined
	Stolfi et al.	SH similar risk of recurrence vs OH	0.17

*HSH* harmonic scalpel haemorrhoidectomy, *OH* open haemorrhoidectomy, *SH* stapled haemorrhoidectomy, *ns* not significant

From 1998 to 2008, SH was associated with less chronic pain postoperatively, with two studies reaching significance. This was mirrored by Ripetti et al. [41] who were able to demonstrate a lower risk of anal stenosis (*p* = 0.004) and chronic rectal pain (*p* < 0.01) with SH in the 2009–2019 group. There was a study that suggested SH had a higher risk of causing postoperative tenesmus (*p* = 0.0012) [43]. As far as other complications were concerned, there were no differences between SH and OH. For recurrence, from 1998 to 2008, close to half of the articles (5 of 14) raised concerns of higher recurrence in SH but only 2 studies reached significance, while from 2009 to 2019, the number was reduced to 2 out of 7 with both studies reaching significance.

### Patient satisfaction

From 1998 to 2008, 22 articles assessed postoperative patient satisfaction. Fourteen of the 22 articles failed to demonstrate a difference in satisfaction scores between SH and OH groups but 6 studies reported statistically significant satisfaction post SH (Table 8).

**Table 7:** Risks of long-term complications in articles published 2009–2019

Complication type	Studies which assessed defined complication	Study conclusions	*p* value
Fistulae	Kim et al.	SH lower risk of fistula formation vs OH	ns
	Ripetti et al.	SH similar risk of fistula formation vs OH/ SCH	0.39
	Bilgin et al.	SH higher risk of fistula formation vs HSH	ns
Incontinence	Wang et al.	SH lower risk of incontinence vs OH	< 0.05
	Kim et al.	SH similar risk of incontinence vs OH	0.559
	Lehur et al.	SH similar risk of incontinence vs HAL	ns
	Ripetti et al.	SH similar risk of incontinence vs OH/ SCH	0.38
	Watson et al.	SH higher risk of incontinence vs OH/ CH	ns
Anal stenosis	Bilgin et al.	SH lower risk of stenosis vs HSH	ns
	Ripetti et al.	SH lower risk of stenosis vs OH	0.004
	Watson et al.	SH higher risk of stenosis vs OH	ns
Chronic pain	Ripetti et al.	SH lower risk of chronic pain vs OH/ SCH	< 0.01
	Lehur et al.	SH similar risk of chronic pain vs HAL	0.87
Tenesmus/urgency	Watson et al.	SH higher risk of tenesmus vs OH/ CH	0.0012
Recurrence	Giarratano et al.	SH lower risk of recurrence vs THD	0.04
	Leung et al.	SH lower risk of recurrence vs THD	< 0.00001
	Kim et al.	SH similar risk of recurrence vs OH	0.65
	Lehur et al.	SH similar risk of recurrence vs HAL	0.65
	Ripetti et al.	SH similar risk of recurrence vs OH/SCH	0.8
	Bilgin et al.	SH higher risk of recurrence vs HSH	< 0.05
	Watson et al.	SH higher risk of recurrence vs OH	< 0.0001

*CH* closed haemorrhoidectomy, *HAL* haemorrhoidal artery ligation, *HSH* harmonic scalpel haemorrhoidectomy, *OH* open haemorrhoidectomy, *SCH* semi-closed haemorrhoidectomy, *SH* stapled haemorrhoidectomy, *THD* transanal haemorrhoidal dearterialization, *ns* not significant

**Table 8:** Articles with patient expressed satisfaction or quality of life scoring post haemorrhoidectomy

Author	*p* value	Article conclusion
**1998–2008**		
Basdanies et al.	Undefined	SH similar to OH
Cheetham et al.	0.76	SH similar to OH
Gravie et al.	Undefined	SH similar to OH
Kairaluoma et al.	0.2	SH similar to OH
Kraemer et al.	1	SH similar to OH
Lai et al.	0.39	SH similar to OH
Mehigan et al.	Undefined	SH similar to OH
Ooi et al.	Undefined	SH similar to OH
Picchio et al.	undefined	SH similar to OH
Smyth et al.	Undefined	SH similar to OH
Wilson et al.*	Undefined	SH similar to OH
Ganio et al.	0.33	SH similar to OH
Palimento et al.	0.735	SH similar to OH
Stolfi et al.	Undefined	SH similar to OH
Bikhchandani et al.	< 0.01	SH superior
Chunget al.	0.001	SH superior
Leventoglu et al.	0.008	SH superior
Wong et al.	0.03	SH superior
Pavlidis et al.	< 0.05	SH superior
Racalbuto	< 0.0001	SH superior
Martinsons et al.*	Undefined	Undefined
Ortiz et al.	Undefined	Undefined
**2009–2019**		
Ammaturo et al.	Undefined	SH similar to OH
Watson et al.*	Undefined	SH similar to OH
Wang et al.	< 0.01	SH superior

*OH* open haemorrhoidectomy, *SH* stapled haemorrhoidectomy

^*^Studies with quality of life assessment without satisfaction scoring

Regarding articles published from 2009 to 2019, Wang et al. compared overall satisfaction scores of SH and OH, and reported a 97% satisfaction rate in SH vs 78% in OH [40]. An analysis of patient quality of life calculated via the EQ 5D 3L score was performed in the eTHOs trial by Watson et al. [43]. They demonstrated that scores were higher in the SH group up to 6 weeks postoperatively (*p* = 0.0235). At 12 months, there was no statistically significant difference in satisfaction between SH and OH or closed haemorrhoidectomy (CH) patient groups, but at 24 months, satisfaction became highest in the OH/CH group (*p* = 0.0342).

### Cost

The cost of treatments was mentioned in three of the studies. In 2002, Wilson et al. calculated that open haemorrhoidectomy cost $1798 per procedure, higher than that of Autosuture stapled anopexy ($1156) and Ethicon stapled anopexy ($1312). In 2016, the eTHOs trial showed that SH cost £941 per patient and OH or CH cost £602 per patient, concluding that SH costs more and provides a lower number of quality-adjusted life years (QALYs) per patient than OH or CH [43]. Bilgin et al. [42] did not perform a formal cost analysis but pointed out that the equipment costs for the harmonic scalpel were double that for SH.

## Discussion

SH was introduced in 1998 as a conceptually attractive surgical technique as it mobilizes the prolapsed rectal mucosa above the dentate line, back to its original anatomical position [48]. Since stapling is meant to be performed above the dentate line on insensate rectal mucosa, postoperative pain and discomfort ought to be minimized, thereby positively influencing length of stay [42]. Resection of a circumferential ring of rectal mucosa eliminates all distal feeding vessels from the superior rectal artery, theoretically attaining a higher degree of surgical completeness and an expected lower risk of recurrence.

This systematic review appears to show that SH is a safe procedure potentially associated with decreased intraoperative blood loss and operative times. It is interesting to note that the shorter SH operative time was already well demonstrated early on (1998–2008) but did not improve further during the latter time period (2009–2019), as shown in Tables 3, 4. In fact, the operative time did not change across the two decades, perhaps indicating that a plateau is reached early beyond which further improvement is not possible [20].

SH seems to be less painful in the immediate postoperative period leading to less urinary retention. In the longer term, although pain was less frequent post SH, overall patient satisfaction appears to decline with time with OH/CH associated with greater quality of life scores after 2 years [43]. Despite the lack of formal statistical analysis when it came to cost-evaluation, mainly due to the fact that cost was not consistently reported in all studies, SH is probably less cost efficient compared to OH or CH, although the supporters of the procedure state that it is not the most resource-demanding procedure on the market and savings are accrued through reduced operative time and shorter length of hospital stay. However, the eTHos trial reported a lower number of QALYs casting further doubts on its potential advantages [43].

Recurrence is an important measure of technical efficacy and SH appears to be just as effective as other surgical interventions. Reports in the past had shown concerns about using SH on grade IV haemorrhoids due to the risk of higher recurrence but this has not been convincingly demonstrated. In our review, the risk of recurrence was deemed similar in SH and OH through both decades, with a smaller fraction of published articles demonstrating higher recurrence risks in SH from 2009 to 2019 compared to 1998–2008, possibly due to increased experience and improved stapling devices. Doppler-guided transanal haemorrhoidal dearterialization (THD) did, however, seem to consistently be associated with higher recurrence rates than SH [45]. Furthermore, despite the inability of SH to excise external haemorrhoidal components, it was often observed that the external lesions shrink due to disruption of their blood supply [34]. It has also been suggested that as the stapling preserves the haemorrhoid tissues and seeks only to disrupt its blood supply, its elimination is more physiological than outright excision, therefore reducing the risk of anal stenosis [12], which supports the findings in Table 7.

Severe complications such as deep pelvic sepsis and peritonitis are recognised but rare complications of haemorrhoidal surgery. It was thought that SH, due to the extent of its tissue manipulation and circumferential involvement of the rectal mucosa, would dispose patients to a higher risk of deep infections. Furthermore, rectal perforation was considered to be an almost exclusive complication of SH from inappropriate deployment of the stapling mechanism [52]. Our review did not identify a single case of these complications although anecdotally they have occurred. Furthermore, it is worthy of note that several other high-volume techniques used in haemorrhoid surgery such as Doppler-guided THD [53] as well as haemorrhoid laser procedure (HeLP) [54] have been associated with postoperative complications necessitating faecal diversion. These occurrences show that the potential for postoperative morbidity is not exclusive to SH. Nonetheless, one must be vigilant in recognizing rare complications such as rectal pocket syndrome [55], rectocoele and rectal intussusception [56] so as to prevent severe pelvic or intra-abdominal sepsis.

Studies were keen to include patients’ self-rated satisfaction levels following surgery in an attempt to quantify the more subjective components of a successful procedure. The most comprehensive of them used the EQ 5D 3L, which captures personal dimensions of mobility, self-care, activity, pain, anxiety as well as an overall self-rated well-being score [57]. SH was good in measurements of patient satisfaction in most selected RCTs we evaluated [40, 47] even when placed under the scrutiny of EQ 5DL 3L, although satisfaction appeared to decline with time [43].

Current evidence suggests that SH is a safe surgical option in the management of haemorrhoid disease. It continues to be embraced by many in the field as a robust and reliable technique with potential, but not fully proven, advantages, at least in the short term. Nevertheless, it has not been found to be inferior to other techniques in this review. The concerns regarding the use of staples remain, which explains why the technique has not been widely adopted in the UK and many other countries. Perhaps the use of biodegradable staples may alleviate some of these concerns and further improve its safety profile.

## Conclusions

The evidence is lacking at the moment to suggest routine use of SH but it can be safely considered in selected patients. It is still not known what group of patients is most likely to benefit from the procedure.
